# Identification of a Conserved Linear Epitope on the p54 Protein of African Swine Fever Virus

**DOI:** 10.3390/v17060823

**Published:** 2025-06-07

**Authors:** Kuijing He, Yue Wu, Zhipeng Su, Yue Zeng, Guishan Ye, Qi Wu, Long Li, Anding Zhang

**Affiliations:** 1 State Key Laboratory of Agricultural Microbiology, Hubei Hongshan Laboratory, College of Veterinary Medicine, Huazhong Agricultural University, Wuhan 430070, China; hekuijing@webmail.hzau.edu.cn (K.H.); yuewu@webmail.hzau.edu.cn (Y.W.); zengyue@webmail.hzau.edu.cn (Y.Z.); ygs@webmail.hzau.edu.cn (G.Y.); wuqi1109323413@webmail.hzau.edu.cn (Q.W.); 2 Key Laboratory of Preventive Veterinary Medicine in Hubei Province, The Cooperative Innovation Center for Sustainable Pig Production, Wuhan 430070, China; 3 Dekon Food and Agriculture Group, Chengdu 610200, China; suzp2023@126.com; 4 Key Laboratory of Development of Veterinary Diagnostic Products, Ministry of Agriculture of the People’s Republic of China, Wuhan 430070, China; 5 International Research Center for Animal Disease, Ministry of Science and Technology of the People’s Republic of China, Wuhan 430070, China; 6 Guangdong Provincial Key Laboratory of Research on the Technology of Pig-Breeding and Pig-Disease Prevention, Guangzhou 510000, China

**Keywords:** monoclonal antibody, African swine fever virus, p54 protein, B-cell epitope, blocking ELISA

## Abstract

African swine fever virus (ASFV) is a highly virulent pathogen that causes nearly 100% mortality in acute infections and poses persistent risks. Effective containment of ASFV outbreaks requires rapid and reliable diagnostic tools. The p54 protein, a key structural component of ASFV, has emerged as an important target for serological detection. Herein, the recombinant p54 protein (amino acids 53–184) was expressed in *Escherichia coli*, and three mouse monoclonal antibodies (mAbs) (IgG1/kappa subtype) were developed. Among these mAbs, the mAb 1F9 specifically recognized the B-cell epitope ^66^IQFINPYQDQQ^76^, which is conserved across different genotypes of ASFV, suggesting that the epitope may serve as a valuable target for serological detection of ASFV. Structural modeling analysis revealed that this epitope is surface-exposed on the p54 protein, with ^67^Gln and ^68^Phe identified as critical residues for 1F9 binding. Moreover, a blocking ELISA based on the mAb 1F9 was established for detecting ASFV-specific antibodies in clinical serum samples, achieving a coincidence rate exceeding 95%. These findings demonstrate that mAb 1F9, targeting a conserved and accessible region of p54, represents a valuable tool for ASFV serodiagnosis, surveillance, and outbreak management.

## 1. Introduction

African swine fever (ASF) is a highly contagious and lethal disease of swine caused by African swine fever virus (ASFV), with case-fatality rates reaching up to 100% in acute infections. ASF has emerged as a major threat to global pig production. Currently, no commercial vaccines or antiviral treatments are available, and the most effective strategy for disease control remains the rapid detection and removal of infected animals [1,2]. Therefore, early and accurate diagnosis is essential for effective ASF prevention and containment.

Current diagnostic approaches for ASFV include nucleic acid detection and serological assays. Recently, the emergence of low-pathogenic ASFV with low viral loads or intermittent shedding may lead to the missed detection of pathogenic nucleic acids [3]. Notably, such infections can still cause seropositivity and serve as persistent sources of virus transmission. Under these circumstances, reliable and timely serological diagnosis becomes critical for effective surveillance and control of ASFV spread [4].

ELISA, a widely applied serological diagnostic method, typically targets the ASFV structural proteins p72 and p30, which are essential components of currently available commercial assays [5,6]. In addition to these, p54, encoded by the *E183L* gene and localized to the virus’s inner envelope, represents another important diagnostic antigen that warrants further evaluation [7]. P54 is classified as a type II membrane protein, featuring a cytoplasmic membrane-anchoring domain and an extracellular domain exposed on the virion surface. It cooperates with p30 to mediate viral attachment to host cells. Once the ASFV enters the cell, p54 interacts with the host cytoplasmic motor protein dynein, participating in the molecular mechanism of intracellular viral transport [8]. Functionally, p54 plays a pivotal role in viral internalization and induces a robust humoral immune response. Antibodies against p54 are typically detectable as early as 8 days post-infection and can persist for several weeks [9,10]. Serological assays utilizing p54 have demonstrated high diagnostic accuracy [11], reinforcing its value as a target for antibody detection. Although mAbs against p54 have been developed in prior studies [11,12,13,14], comprehensive mapping of its immunodominant epitopes remains limited. Further characterization of these epitopes is essential to improve diagnostic tools and deepen our understanding of host–virus interactions.

In this study, the p54 protein was efficiently expressed and purified, and mAbs against p54 were generated via hybridoma screening. Furthermore, the B-cell epitope recognized by one of the mAbs was highly conserved among ASFV strains. By targeting this conserved epitope, the selected mAb offers enhanced diagnostic specificity and reliability. These findings support the development of a robust serological tool for ASFV surveillance and contribute to improved strategies for disease control in affected swine populations.

## 2. Materials and Methods

### 2.1. Expression of the p54 Protein

**Figure 1:**
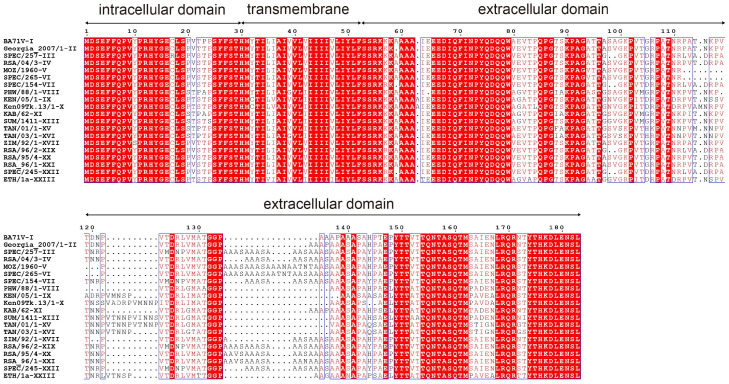
Conservation analysis of the p54 protein. The amino acid sequences of the p54 protein from 22 ASFV genotypes were aligned. The extracellular domain of the p54 protein from the ASFV-Georgia 2007/1 strain was selected for recombinant expression in this study.

The ASFV *E183L* gene sequences from 22 distinct genotypes were retrieved from the NCBI database to evaluate sequence conservation (Figure 1). The extracellular domain sequence of the *E183L* gene from the ASFV-Gerogia 2007/1 strain (GenBank: FR682468.2) was selected for recombinant expression. The gene was synthesized and codon-optimized (Table S1) for expression in *Escherichia coli* by Beijing Tsingke Biotech Co., Ltd. (Beijing, China), and subsequently cloned into the pET-23a vector. The construction was transformed into *E. coli* BL21(DE3) cells, and protein expression was induced with Isopropyl β-D-1-thiogalactopyranoside (IPTG). The recombinant p54 protein was purified using a His-Bind Purification Kit (Ni Sepharose 6 Fast Flow; Cytiva, Shanghai, China), and its concentration was determined with a BCA protein assay kit (Beyotime Biotechnology, Shanghai, China). The purified p54 protein was coated onto plates at the concentration of 1.5 μg/mL for indirect ELISA to detect ASFV-specific antibodies in pig sera, with HRP-conjugated anti-porcine IgG (SouthernBiotech, Beijing, China, 1:10,000) as the secondary antibody. Absorbance was read at 450 nm after TMB reaction termination with 2 M sulfuric acid.

### 2.2. Animal Immunization of Strategy

BALB/c mice (6–8-week-old) were subcutaneously immunized with 100 μg of the p54 protein emulsified with the Freund’s complete adjuvant (Sigma, Shanghai, China). Booster immunizations using the same dose of the antigen were administered on days 14 and 28. Blood samples were collected on days 14, 28, and 42, to assess antibody titers via indirect ELISA. The mice that exhibited the highest antibody response received three intraperitoneal doses of 50 μg of the p54 protein over 6 days as a final boost prior to hybridoma generation.

### 2.3. Preparation of mAbs

Spleen cells from the immunized mice were fused with SP2/0 myeloma cells, using standard polyethylene glycol-mediated fusion. Hybridoma cells were selected and cultured in hypoxanthine-aminopterin-thymidine (HAT) medium (Sigma). Positive clones secreting anti-p54 antibodies were identified by indirect ELISA. To obtain large quantities of mAbs, selected hybridoma cells were injected intraperitoneally into BALB/c mice for ascites production. The mAbs were subsequently purified from ascitic fluid using protein G affinity chromatography (GE Healthcare, Shanghai, China). Finally, a test was performed to identify the type of mAbs created using a commercial isotyping kit (SouthernBiotech).

### 2.4. Western Blotting Analysis

Western blotting analysis was employed to evaluate the reactivity of recombinant p54 protein with different antibodies. The purified p54 protein was denatured by heating at 98 °C for 10 min, separated on a 12% SDS-PAGE gel, and then transferred onto a 0.4-µm nitrocellulose (NC) membrane (Cytiva). Subsequently, it was then incubated at 37 °C for 1 h with ASFV-positive (1:100) and negative sera as primary antibody, followed by incubation with HRP-conjugated anti-porcine IgG (Southern Biotech, 1:10,000). Signal detection was performed using the ChemiDoc Touch system (BIO-RAD, Beijing, China).

To assess the specificity of mAbs, Western blotting analysis was performed using both the p54 protein and ASFV-infected primary porcine alveolar macrophages. Briefly, PAMs were cultured in 12-well plates, infected with ASFV, and harvested 24 h post-infection. The cell lysates and the p54 protein were denatured by heating at 98 °C for 10 min and separated on a 12% SDS-PAGE gel. The proteins were then transferred to 0.4-µm nitrocellulose membranes (Cytiva), followed by incubation with individual mAbs to assess antigen recognition.

### 2.5. Indirect Immunofluorescence Assay (IFA)

An IFA was performed to evaluate the specificity of the anti-p54 mAbs. For the IFA, ASFV-infected PAMs were treated with a mixture of cold acetone and methanol for 20 min. To permeabilize the cells and facilitate antibody entry, 0.2% Triton X-100 was applied for 15 min, and then three washes with PBS were performed. Non-specific binding was blocked by incubating cells with 10% goat serum for 1.5 h at room temperature. After that, the cells were kept at 37 °C for 1 h with anti-p54 mAbs and washed with PBS. The FITC-conjugated goat anti-mouse IgG (Beyotime, Shanghai, China) was used as a maker, and the cells were mounted using an antifading medium containing DAPI and examined using fluorescence microscopy(MSHOT, Guangzhou, China).

### 2.6. Identification of Linear B-Cell Epitopes

To identify the linear B-cell epitope recognized by mAbs, superfolder GFP-tagged (sfGFP-tagged) fragments of the p54 protein were expressed in *E. coli* following induction with 0.8 mM IPTG. The full-length p54 protein served as a positive control, while sfGFP protein alone was used as a negative control. Western blotting analysis was performed using hybridoma supernatants as the primary antibody source to evaluate the reactivity between mAbs and the p54 protein fragments. Stepwise truncation of the p54 protein sequence allowed the identification of the minimal linear epitope recognized by the mAbs.

### 2.7. Blocking ELISA Analysis of ASFV-Positive Sera

A blocking ELISA was developed for detecting ASFV antibodies in serum samples. MAb 1F9 was purified using protein G resin and conjugated with HRP following kit instructions; a checkerboard ELISA was then performed to determine the optimal concentrations of the coated p54 protein and HRP-conjugated 1F9 for use in the assay. The recombinant p54 protein (0.6 μg/mL) was coated onto 96-well plates using carbonate buffer and incubated overnight at 4 °C. Then the plates were treated with 5% BSA at 37 °C for 1 h. ASFV positive and negative serum samples were diluted 1:2 and incubated at 37 °C for 30 min. After washing, HRP-labeled mAb 1F9 (1:100) was added to each well and incubated at 37 °C for 1 h. Absorbance was read at 450 nm after TMB reaction termination with 2 M sulfuric acid (H_2_SO_4_). The percentage inhibition (PI) was calculated using the formula: PI (%) = [1 − (OD_450nm_ of test sample/OD_450nm_ of negative control)] × 100%.

### 2.8. Analysis of the Spatial Structure of Epitopes

The 3D model of the p54 protein and its antigen–antibody interaction interface were predicted using AlphaFlod3 (https://alphafoldserver.com/), and PyMOL software (version 2.5.2, DeLano Scientific LLC, San Carlos, CA, USA) was employed to visualize the binding sites.

## 3. Results

### 3.1. The Generation of the Recombinant p54 Protein

**Figure 2:**
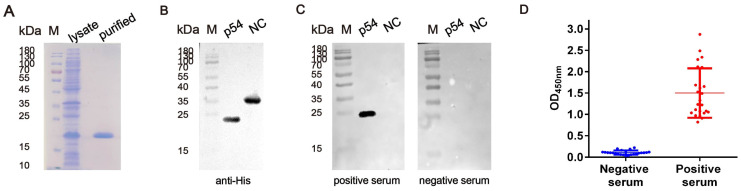
Expression and activity verification of the p54 Protein. (**A**) Expression and purification of p54 protein. M: protein marker; lysate: supernatant after induction and cell lysis; purified: purified p54 protein. (**B**,**C**) Western blotting analysis of the purified p54 protein. The protein was detected using an anti-His tag mAb, ASFV-positive pig sera, and ASFV-negative pig serum. M: protein marker; p54: purified p54 protein; NC: an irrelevant pET-23a overexpressed protein. (**D**) Antigenicity evaluation of the p54 protein. Indirect ELISA showed specific reactivity of the p54 protein with ASFV-positive serum samples (*n* = 24, diluted 1:100), while no reactivity was observed with negative serum samples (*n* = 24, diluted 1:100). The optimal coating concentration of the p54 protein in ELISA was determined to be 1.5 μg/mL.

Based on transmembrane domain analysis of the p54 protein sequence, the gene encoding its extracellular domain was cloned and expressed recombinantly in *E. coli* BL21 (DE3) cells. Using a His-tag purification kit, the protein was purified to high purity, with an approximate molecular weight of 20 kDa (Figure 2A,B). Western blotting analysis further verified the specific reactivity of the p54 protein with ASFV-positive sera (Figure 2C). Additionally, indirect ELISA results demonstrated strong reactivity with ASFV-positive sera and no detectable signal with ASFV-negative sera (Figure 2D). These results indicated that the recombinant p54 protein retains strong immunoreactivity and can specifically recognize ASFV-positive sera.

### 3.2. Generation and Characterization of Anti-p54 mAbs

**Figure 3:**
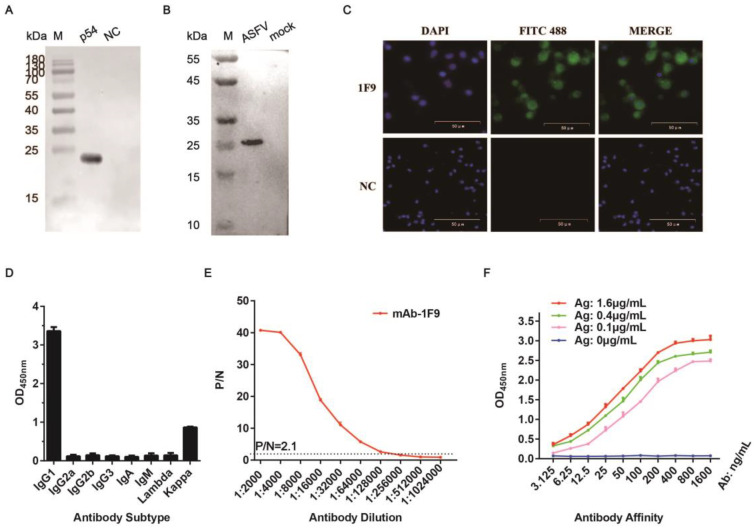
Characterization and identification of the 1F9 mAb. (**A**) Specific recognition of the recombinant p54 protein by the 1F9 mAb. M: protein marker; p54: purified p54 protein; NC: an irrelevant pET-23a overexpressed protein. (**B**) Detection of the p54 protein expressed in ASFV-infected PAMs using the 1F9 mAb. M: protein marker; ASFV: ASFV-infected PAMs; mock: uninfected PAMs as negative control. (**C**) IFA demonstrated that the reactivity of mAb 1F9 against ASFV-infected PAMs. (**D**) Identification of the subclass of 1F9 mAb. (**E**) Titer determination of mAb 1F9 by indirect ELISA. P/N value ≥ 2.1 was defined as positive, and the highest dilution satisfying this threshold was defined as the antibody titer. (**F**) Affinity determination of 1F9 mAb.

Three hybridoma cell clones (1F9, 1B4, and 5F10) were selected using standard hybridoma technology, and these clones stably secrete p54-specific mAbs. Western blotting analysis demonstrated all these three mAbs recognized both recombinant p54 protein and ASFV-infected PAMs (Figure 3A,B and Figure S1A,B), indicating that they target linear epitopes of p54. The IFA analysis further confirmed that all three mAbs specifically recognized ASFV-infected PAMs (Figure 3C and Figure S1C). Isotype analysis revealed that all mAbs belonged to the IgG1 subclass and contained Kappa light chains (Figure 3D and Figure S1D). ELISA titration indicated that the antibody titers of mAbs 1F9, 1B4, and 5F10 were 1:128,000, 1:512,000, and 1:256,000, respectively (Figure 3E and Figure S1E). The affinity constant of mAb 1F9 was determined using three concentrations of the p54 antigen (1.6, 0.4, and 0.1 μg/mL), with the corresponding EC50 values calculated as 0.03306, 0.04126, and 0.07089 μg/mL, respectively (Figure 3F). The affinity constant of mAbs 1B4 and 5F10 were calculated using antigen concentrations of 3.2, 0.8, and 0.2 μg/mL. The corresponding EC50 values of the mAb 1B4 were 0.03498, 0.04242, and 0.06244 μg/mL, while those for mAb 5F10 were 0.06779, 0.12488, and 0.14531 μg/mL (Figure S1F). Sequencing of the variable regions of the mAbs 1F9, 1B4 and 5F10 hybridoma cells confirmed their murine origin and alignment with the BALB/c IgG sequences. Critical amino acid residues within the complementarity-determining regions (CDRs) were highlighted in red, supporting their structural integrity and specificity for antigen recognition (Figure S2).

### 3.3. Identifying the Epitope Recognized by the p54 mAbs

**Figure 4:**
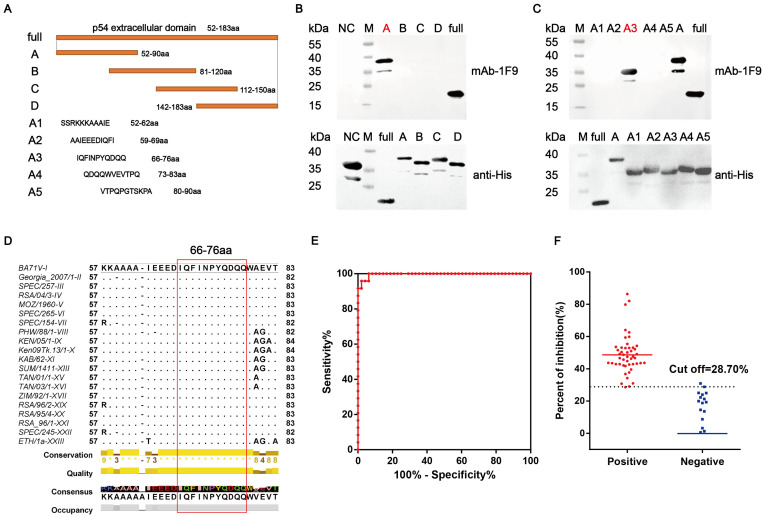
Epitope mapping of p54 and establishment of a blocking ELISA. (**A**) Schematic representation of the p54 truncated overlapping peptide segments. (**B**) Preliminary epitope mapping of 1F9 using Western blotting analysis. (**C**) Fine epitope mapping of 1F9. Peptides A–D and A1–A5 were cloned into the pET-23a plasmid and expressed as sfGFP-His-tag fusion proteins in *E. coli*. The truncated peptides were analyzed via Western blotting using the 1F9 mAb. M: protein marker. NC: negative control (pet-23a-sfGFP). (**D**) Sequence alignment of p54 from 22 ASFV genotypes using Jalview. The identified epitope region (aa 66–76) recognized by 1F9 is highlighted in red. The consensus sequence, ^66^IQFINPYQDQQ^76^, was conserved across ASFV genotypes. (**E**) Blocking ELISA analysis using ASFV-positive (*n* = 48) and ASFV-negative (*n* = 24) serum samples. The receiver operating characteristic (ROC) analysis yielded an area under the curve (AUC) value of 0.9967, demonstrating high test accuracy. (**F**) Dynamic dot plot showing the blocking values of serum samples. The cutoff value for positiveness was set at 28.70%.

The IEDB (http://tools.iedb.org/bcell/, accessed on 6 July 2022) was utilized to predict potential B-cell epitopes on the p54 protein, highlighting three candidate regions in yellow (Figure S3A). To identify the B-cell epitope recognized by all mAbs, the extracellular domain of the p54 protein was split into four sections that overlap each other. Each segment was fused to the sfGFP gene and cloned into the pET-23a vector (Figure 4A and Figure S3B). The expressed fusion proteins, designated p54-A, p54-B, p54-C, and p54-D, were produced in *E. coil* and analyzed for mAbs recognition via Western blotting. The results demonstrated that mAb 1F9 specifically recognized amino acids (aa) 53–91 of the p54-A segment (Figure 4B). Additionally, the mAbs 1B4 and 5F10 specifically recognized the p54-D fragment (aa 142–183; Figure S3B). To further refine the binding site for mAb 1F9, the p54-A sequence was subdivided into five overlapping fragments, A1, A2, A3, A4, and A5 (Figure 4A). Western blotting analysis revealed that mAb 1F9 exclusively recognized the A3 segment, corresponding to residues ^66^IQFINPYQDQQ^76^ (Figure 4B,C). Similarly, the specific epitopes of 1B4 and 5F10 were mapped to p54-D3 (^158^ASQTMSAIENLR^169^) and p54-D5(^173^TYTHKDLENSL^183^), respectively (Figure S3C,D).

To evaluate the conservation of the identified epitope regions among different ASFV genotypes, the p54 protein sequences from 22 ASFV genotypes available in the NCBI database were analyzed. Alignment demonstrated high conservation of the identified epitope sequences across isolates of mAb 1F9 (Figure 4D). In contrast, the epitopes targeted by mAbs 1B4 and 5F10 exhibited sequence variability across different ASFV strains (Figure S3F). Based on this high degree of conservation, mAb 1F9 was selected for the development of a serological assay targeting the p54 protein.

A blocking ELISA was developed using the p54 protein in combination with ASFV-positive and -negative sera, and an HRP-conjugated mAb 1F9. To evaluate the diagnostic performance of the assay, a total of 72 swine serum samples were tested (including 24 ASFV-negative and 48 ASFV-positive samples). The assay yielded an area under the ROC curve (AUC) of 0.9965 (95% CI: 98.92 to 100.4%), with an optimal cut-off value determined at 28.70% inhibition (Figure 4E), indicating excellent diagnostic accuracy. Furthermore, the dot plot distribution of inhibition rates clearly illustrated the established cut-off and enabled distinct differentiation between positive and negative samples, reinforcing the assay’s reliability (Figure 4F).

### 3.4. Key Residues of Epitope for Recognition by p54 mAb

**Figure 5:**
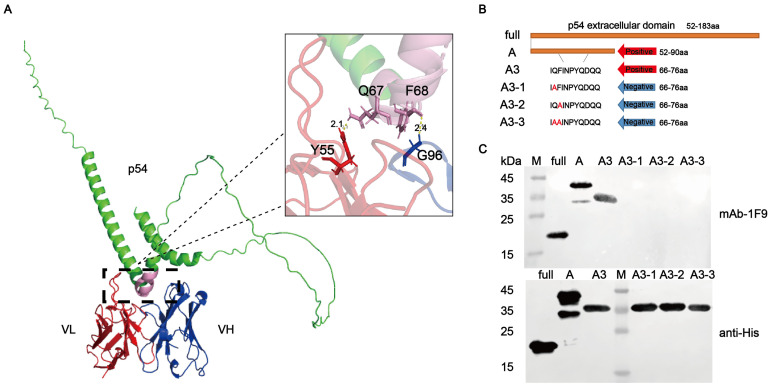
Identification of linear epitopes and key amino acid residues recognized by the 1F9 mAb. (**A**) Structural prediction of the p54 protein binding to the 1F9 antibody. The structural model was generated using AlphaFlod3 and analyzed with PyMOL to identify the binding epitope region on the p54 protein and predict key amino acid residues involved in antibody recognition. (**B**) Schematic diagram of p54 truncated mutant peptides used for epitope mapping. (**C**) Functional validation of critical binding residues Q67 and F68. Peptides A3-1, A3-2, and A3-3 were cloned into the pET-23a plasmid, expressed as sfGFP-His-tag fusion proteins in *E. coli*, and analyzed via Western blotting using the 1F9 mAb. M: protein marker.

The antigenic epitopes recognized by the mAb 1F9 were predicted using AlphaFold3 and visualized with PyMOL. Structural analysis indicated that the epitope recognized by 1F9 is located on the surface of the p54 protein (Figure 5A), suggesting high accessibility and favorable conditions for antibody binding. Key binding residues, Q67 and F68, were identified as critical sites for antigen–antibody interaction. To validate their functional importance, three mutant peptides A3-1, A3-2, and A3-3 were generated by site-directed mutagenesis and cloned into the recombinant expression plasmid SFGFP-pET-23a (Figure 5B). Western blotting analysis showed that the 1F9 mAb failed to react with the mutant peptides (Figure 5C and Figure S4), confirming that Q67 and F68 are essential for epitope recognition. These findings demonstrate that the identified epitope is highly conserved among various ASFV strains, underscoring its potential as a reliable target for ASFV-specific serological diagnostics.

## 4. Discussion

The absence of an effective vaccine against ASF underscores the urgent necessity for precise and universal diagnostic tools to control viral spread [2]. Among ASFV antigens, the p54 protein is a key target due to its high immunogenicity and essential role in viral infection [15,16]. While previous studies have identified B-cell epitopes of p54 [12,17,18], the conservation and structural accessibility of these epitopes across diverse ASFV genotypes remain underexplored. This study addresses this gap by generating mAbs targeting a conserved immunodominant epitope of p54 and validating its diagnostic potential.

The mAb 1F9 specifically recognizes the linear epitope ^66^IQFINPYQDQQ^76^, which is 100% conserved across all 22 ASFV genotypes available in the NCBI database. This strict conservation suggests strong evolutionary constraints, likely linked to p54’s indispensable role in viral assembly or host interactions. The epitope partially overlaps with a region previously reported to bind antibodies [11,12], suggesting that this motif may represent key immunodominant region of the p54 protein. Furthermore, by integrating structural modeling with mutational analysis, we identified Gln⁶⁷ and Phe⁶⁸ as critical residues involved in antibody binding, thereby enhancing the understanding of antigen–epitope interactions within this region. Importantly, this sequence invariance ensures that mAb 1F9 can detect all known ASFV strains, bypassing challenges posed by genetic variability. Experimental validation using blocking ELISA demonstrated its ability to clearly distinguish ASFV-positive and negative sera, underscoring its reliability as a universal diagnostic reagent.

Structural mapping of the p54 protein (Figure 1) revealed distinct antigenic domains: while prior studies utilized the N-terminal region (aa 1–29) for mAb development [18], we targeted the antibody recognition-enhanced C-terminal domain (aa 53–184). Within this domain, epitope ^66^IQFINPYQDQQ^76^—recognized by mAb 1F9 was found to adopt surface-exposed mixed secondary structures, with structural modeling confirming its positioning within a solvent-accessible loop bridging α-helical and β-sheet motifs on the viral intima protein. The epitope’s exposure on the membrane surface minimizes steric hindrance and maximizes accessibility for antibody binding [8,19], as evidenced by the strong reactivity of mAb 1F9 in immunofluorescence assays (IFA) with ASFV-infected PAMs. This spatial positioning, combined with the epitope’s conserved sequence, ensures high diagnostic sensitivity even in complex biological samples [20].

In this study, a blocking ELISA based on the mAb 1F9 was successfully established. Compared with indirect or competitive ELISA formats, the blocking ELISA offers improved specificity and is less affected by non-specific background signals, making it more suitable for detecting specific antibodies in complex clinical samples. The use of a mAb 1F9 targeting a highly conserved epitope (aa 66–76) ensures broad genotype coverage, reducing the risk of false negatives due to ASFV genetic variability. Preliminary validation of the method showed low background interference and good antigen–antibody binding activity, indicating its potential utility in serological surveillance. However, further evaluation using larger clinical serum samples is necessary to comprehensively assess its diagnostic performance and practical applicability under field conditions.

## 5. Conclusions

This study successfully generated three mAbs against the ASFV p54 protein, all of which specifically recognized native viral antigens in infected cells. The mAb 1F9, targeting the conserved and surface-exposed ^66^IQFINPYQDQQ^76^ epitope, represents a significant advancement in ASFV diagnostics. Its pan-genotypic reactivity eliminates the risk of false negatives caused by genetic diversity, while its structural accessibility ensures robust detection efficiency. The established blocking ELISA protocol provides a scalable and cost-effective tool for large-scale serological screening, directly supporting ASFV surveillance efforts. Future research should focus on further characterizing the functional role of this epitope in the viral life cycle and optimizing its integration into field-deployable diagnostic platforms.

## Data Availability

Data will be made available on request.

## Supplementary Materials

The following supporting information can be downloaded at: https://www.mdpi.com/article/10.3390/v17060823/s1, Figure S1: Characterization and identification of 1B4 and 5F10 mAbs; Figure S2: Sequence of the variable regions of antibody genes; Figure S3: Design of truncated overlapping peptides and epitope identification for 1B4 and 5F10 monoclonal antibodies; Figure S4: Identification of core residues; Table S1: The p54 protein sequence expressed in E. coli..
