# Supplementary material for: Identification of a Conserved Linear Epitope on the p54 Protein of African Swine Fever Virus
Source: Viruses. 2025 Jun 7;17(6):823. doi: 10.3390/v17060823 (PMC12197541; doi:10.3390/v17060823)
Supplement: Supplementary file 1 [file viruses-17-00823-s001.zip › viruses-3609299-supplementary.pdf]

Supporting Information

Development and Characterization of a Monoclonal Antibody Targeting the Conserved Immunodominant Epitope of the p54 Protein in African Swine Fever Virus

Kuijing He[1, 2], Yue Wu[1, 2], Yue zeng[1, 2], Guishan Ye[1, 2], Qi Wu[1, 2], Long Li[1, 2, 3, 4 *], Anding Zhang[1, 2, 3, 4, 5 *]

[1] National Key Laboratory of Agricultural Microbiology, Hubei Hongshan Laboratory, College of Veterinary Medicine, Huazhong Agricultural University, Wuhan, Hubei 430070, China.

[2] Key Laboratory of Preventive Veterinary Medicine in Hubei Province, The Cooperative Innovation Center for Sustainable Pig Production, Wuhan, Hubei 430070, China.

[3] Key Laboratory of Development of Veterinary Diagnostic Products, Ministry of Agriculture of the People's Republic of China, Wuhan, Hubei 430070, China.

[4] International Research Center for Animal Disease, Ministry of Science and Technology of the People's Republic of China, Wuhan, Hubei 430070, China.

[5] Guangdong Provincial Key Laboratory of Research on the Technology of Pig-breeding and Pig-disease prevention, Guangzhou, Guangdong, 510000, China.

---

[*] Corresponding author.

E-mail address: lilong@mail.hzau.edu.cn (Long Li) and andye8019@mail.hzau.edu.cn (Anding Zhang),
Long Li and Anding Zhang contributed equally to this manuscript..

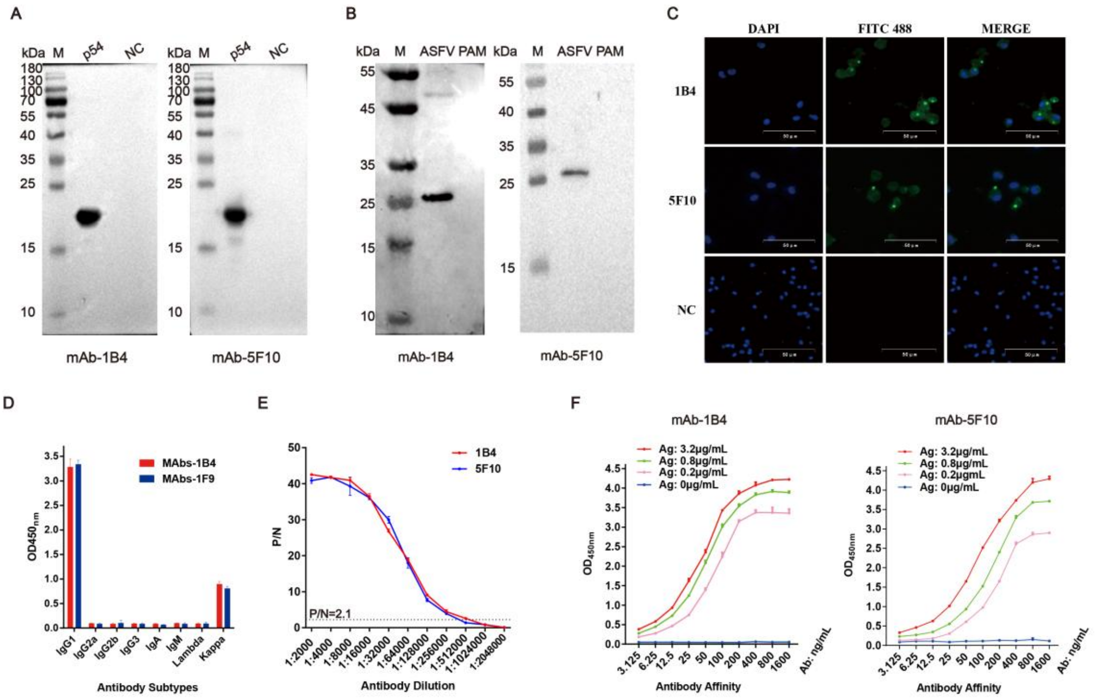

Figure S1 Characterization and identification of 1B4 and 5F10 mAbs. (A) Specific recognition of recombinant p54 protein by 1B4 and 5F10 mAbs. M: protein marker; p54: purified p54 protein; NC: negative control (pET-23a vector without inset). **(B)** Detection of the p54 protein expressed in ASFV-infected PAM cells using 1B4 and 5F10 mAbs. M: protein marker; ASFV: PAM cells infected with ASFV; MOCK: uninfected PAM cells as control. **(C)** Reactivity of monoclonal antibodies in indirect immunofluorescence assay (IFA). **(D)** Subclass identification of 1B4 and 5F10 mAbs. **(E)** Determination of the mAbs titers. P/N value ≥ 2.1 was defined as positive, and the highest dilution meeting this criterion was recorded as the antibody titer. **F** Affinity determination of monoclonal antibodies.

mAb-1F9

VL

FR1 · CDR1 · FR2
NIMMTQSPSSLAVSAGEKVTMSCKSS**QSVLFSSNQKNY**LAWYQQKPGQSPKLLIY

CDR2 · FR3 · CDR3 · FR4
WASTRGSGVPDRFTGSGSGTDFTLTISNVQAEDLAVYYC**HQYLSSYT**FGGGTKLEIK

VH

FR1 · CDR1 · FR2 · CDR2
QVQLQQSGPELVKPGASVRISCKAS**GYTFANYY**LHWMKQRPGQGLEWIGW**IYPG**

FR3 · CDR3 · FR4
NANTEYNEKFKGKATLTADKSSSTAYMQLSSLTSEDSAVYFC**ARTGSMDY**WGQGTT

mAb-1B4

VL

FR1 · CDR1 · FR2 · CDR2
DIELTQSPAIMSASPGEKVTMTCSAS**SSVSY**MHWYQQKPGSSPRLWIY**LTF**KLA

FR3 · CDR3 · FR4
SGVPARFSGSGSGTSYSLSISSMEAEDAATYYW**QQWSSNPWT**FGGGTKLEIK

VH

FR1 · CDR1 · FR2 · CDR2
QVQLQQSGPELVKPGASVKMSCKAS**GYTFNDYV**ISWVKQRTGQGLEWIGE**IYPGNNSS**

FR3 · CDR3 · FR4
YYNEKFKGKATLTADKSSNTAYMQLSSLTSEDSAVYFC**ARSDYYGSILFAH**WGQGTTVTVSS

mAb-5F10

VL

FR1 · CDR1 · FR2 · CDR2
DIVMTQSPSSLTVTAGEKVTLGCKSS**RSLLNSGNQKNY**LTYYQQKPGQPPKLMIY**WAS**

FR3 · CDR3 · FR4
TRESGVPDRFTGSGSGTDFTLTIRSVQAEDLAVYYC**QNDYSYPH**VRCWDQAGAE

VH

FR1 · CDR1 · FR2 · CDR2
QVKPQQSGPSLVKPSQTLSLTCSVT**GDSITSGY**WNWIRKFPGNKLEFMGS**ISYSGNT**

FR3 · CDR3 · FR4
YCHPSLKSRISITRDTSKNQCYLQLNSVTSEDSATYYC**ARTLLYFDV**WGQGTTVNRLL

Figure S2 Sequence of the variable regions of antibody genes. Complementarity-determining regions (CDRs) are highlighted in red, and framework regions (FRs) are shown in black.

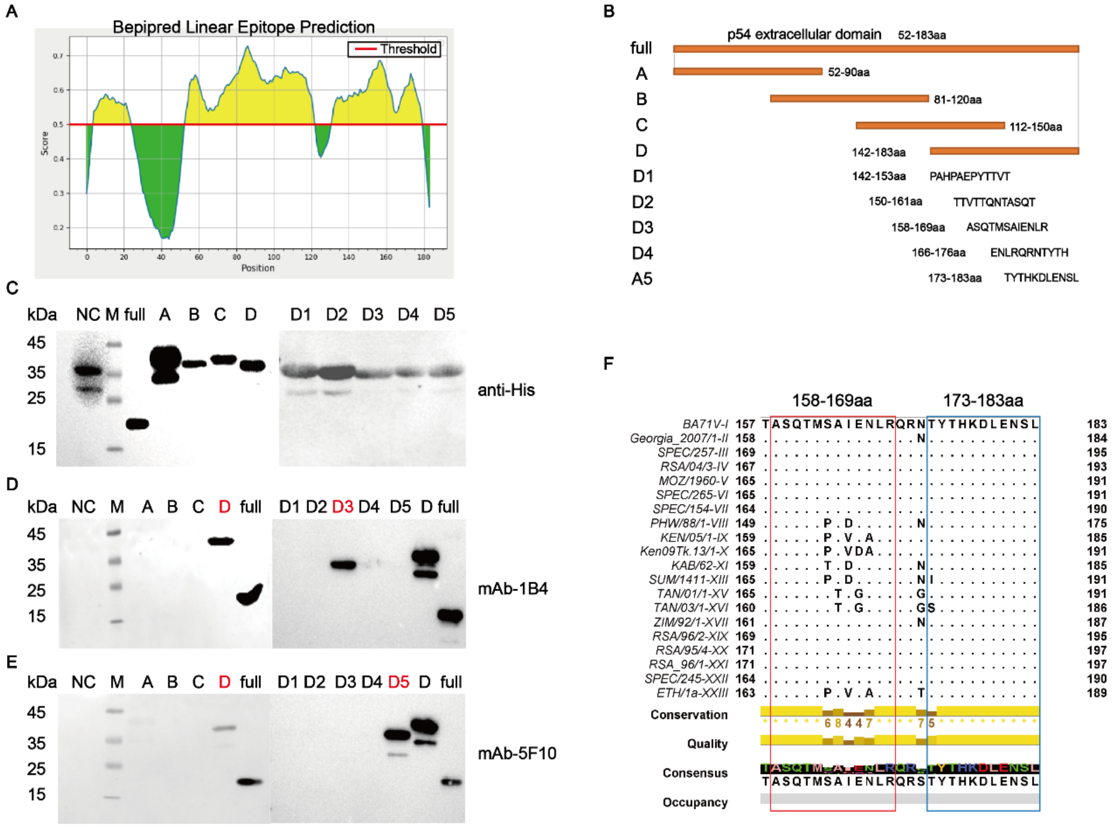

Figure S3 Design of truncated overlapping peptides and epitope identification for 1B4 and 5F10 monoclonal antibodies. (A) Prediction of the B-cell epitopes of ASFV p54 using IEDB. The yellow region is the predicted epitope region. **(B)** Schematic representation of p54 truncated overlapping peptide segments. **(C-E)** Epitope mapping for the 1B4 and 5F10 monoclonal antibodies. Western blot analysis was used to identify precise epitope regions. Peptides A–D and D1–D5 were cloned into the pET-23a plasmid, expressed as sf-GFP-His-tag fusion proteins in E. coli, and tested using the 1B4 and 5F10 monoclonal antibodies. M: protein marker. NC: negative control (pet-23a-sfGFP). (**F**) Sequence alignment of p54 from 22 ASFV genotypes using Jalview. The identified epitope region for 1B4 (158–169 aa) is highlighted in red, with the consensus sequence ASQTMSAIENLR, and the region for 5F10 (173–183 aa) is highlighted in blue, with the consensus sequence TYTHKDLENSL. Both sequences showed variability among

different ASFV genotypes.

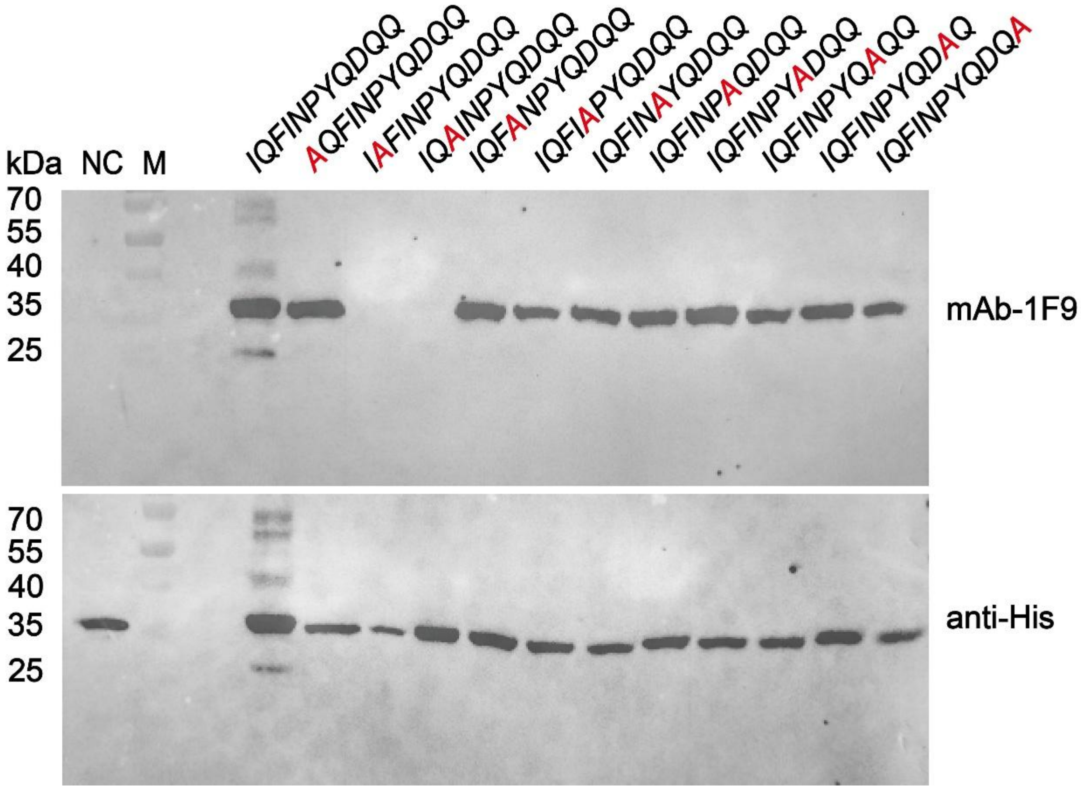

Figure S4. Identification of core residues.
